# Cardiogenic Shock (SCAI Stage D) Following Pharmacological Reperfusion of Anterior ST-Segment Elevation Myocardial Infarction in a Young Woman: A Case Report

**DOI:** 10.7759/cureus.105095

**Published:** 2026-03-12

**Authors:** José I Torres-Verduzco, José I Balderas-Santoyo, Quitzia L Torres-Salazar

**Affiliations:** 1 Internal Medicine, Hospital General de Zona No. 1, Mexican Social Security Institute (IMSS), Durango, MEX; 2 Cardiology, Hospital Regional de Alta Especialidad, Institute for Social Security and Services for State Workers (ISSSTE), Veracruz, MEX; 3 Biomedical Sciences, Universidad Juárez del Estado de Durango, Durango, MEX

**Keywords:** anterior myocardial infarction, cardiogenic shock, pharmacological reperfusion, st-segment elevation myocardial infarction, young patient

## Abstract

Cardiogenic shock remains the leading cause of mortality in patients with ST-segment elevation myocardial infarction, typically occurring in older individuals with established cardiovascular disease. However, severe hemodynamic deterioration may also develop in younger patients outside the expected epidemiological profile. We report the case of a 39-year-old woman who presented with an anterior ST-segment elevation myocardial infarction and subsequently developed cardiogenic shock following pharmacological reperfusion. Despite the absence of traditional cardiovascular risk factors, the patient experienced rapid clinical deterioration requiring vasoactive and inotropic support. Electrocardiographic findings demonstrated extensive anterior and anteroseptal myocardial involvement, highlighting infarct size and location as key determinants of shock severity. The clinical course illustrated the dynamic nature of cardiogenic shock, which evolved during hospitalization rather than at initial presentation. Application of the Society for Cardiovascular Angiography and Interventions (SCAI) classification provided a useful framework for early recognition and management escalation. This case emphasizes that younger age does not preclude the development of cardiogenic shock and underscores the importance of close post-reperfusion monitoring and physiology-oriented clinical assessment beyond age-based risk stratification.

## Introduction

Cardiogenic shock remains the leading cause of in-hospital mortality among patients with acute myocardial infarction despite advances in reperfusion strategies and critical care management. It results from inadequate tissue perfusion due to primary cardiac dysfunction and occurs in approximately 5-10% of patients with acute myocardial infarction, with short-term mortality approaching 40% and one-year mortality nearing 50%. The most common mechanism is extensive myocardial ischemia leading to severe impairment of ventricular contractility, most frequently associated with anterior wall infarction involving the left anterior descending coronary artery [1,2].

Although cardiogenic shock is typically reported in older patients with pre-existing cardiovascular disease and multiple risk factors, it may also occur in younger individuals without previously documented structural heart disease. In these cases, the condition may fall outside the expected epidemiological profile, challenging age-based assumptions and emphasizing the need for clinical vigilance beyond chronological risk stratification. Moreover, cardiogenic shock may arise at presentation or develop dynamically during hospitalization, including after reperfusion therapy, reflecting the complex interplay between myocardial necrosis, inflammatory responses, and acute ventricular dysfunction rather than delayed reperfusion alone [2,3].

The Society for Cardiovascular Angiography and Interventions (SCAI) proposed a classification system that conceptualizes cardiogenic shock as a clinical spectrum, facilitating early recognition and escalation of hemodynamic support through serial assessment [4]. Despite contemporary management based on rapid reperfusion, vasoactive therapy, and intensive monitoring, outcomes remain poor and may be influenced by infarct extent and systemic factors. We report a case of cardiogenic shock that developed after pharmacological reperfusion of an anterior ST-segment elevation myocardial infarction in a young woman without known cardiovascular disease, illustrating the dynamic nature of shock, challenging age-based risk assumptions, and highlighting the value of structured physiological assessment. The case is reported in accordance with the Surgical Case Report (SCARE) 2025 guidelines [5].

## Case presentation

A 39-year-old woman was admitted to the emergency department due to acute-onset chest pain. She was originally from and resided in San Juan del Río, Durango, Mexico, worked as a merchant, and had completed primary education. Prior to admission, she was independent in activities of daily living and instrumental activities.

Her past medical history was significant for hyperthyroidism treated with total thyroidectomy in 2021, complicated by secondary hypoparathyroidism. She was receiving levothyroxine 100 μg once daily. Calcium supplementation had been previously prescribed but was discontinued in 2023 following medical advice. There was no history of hypertension, diabetes mellitus, dyslipidemia, smoking, alcohol consumption, illicit drug use, or previously diagnosed cardiovascular disease. Family history was noncontributory.

At presentation, the patient reported sudden-onset retrosternal chest pain that developed while at rest. The pain was described as oppressive, with severe intensity (10/10), radiating to the left upper limb, without identifiable relieving or aggravating factors. The chest pain was accompanied by generalized diaphoresis, nausea, and two episodes of vomiting of gastric content, prompting medical evaluation.

On arrival, vital signs showed a blood pressure of 107/67 mmHg, heart rate of 86 beats per minute, respiratory rate of 20 breaths per minute, and temperature of 36.7°C. During hospitalization, she was admitted to the internal medicine service, where vital signs demonstrated blood pressure of 93/61 mmHg (mean arterial pressure 71 mmHg), heart rate of 97 beats per minute, respiratory rate of 20 breaths per minute, temperature of 36.5°C, and oxygen saturation of 95% while receiving supplemental oxygen via a nonrebreather mask at 8 L/min (FiO₂ 60%).

Physical examination revealed a woman appearing her stated age, alert and oriented, cooperative, with pallor of the skin and conjunctivae, and no evidence of jaundice. Cardiovascular examination demonstrated rhythmic heart sounds with normal intensity, without murmurs or additional sounds. Pulmonary examination revealed clear lung fields without crackles or wheezes. There was no jugular venous distension, peripheral edema, or signs of volume overload. The abdomen was soft, nontender, and without organomegaly. Neurological examination was unremarkable.

Initial laboratory evaluation revealed leukocytosis, normal hemoglobin concentration, preserved renal function, and elevated serum glucose. Serum electrolytes showed low-normal total calcium levels, with normal magnesium and phosphorus concentrations at presentation. Inflammatory markers were within normal limits. Initial cardiac biomarkers demonstrated a minimally elevated troponin level.

During hospitalization, arterial blood gas analysis demonstrated hypoxemia with preserved acid-base balance. Serial laboratory studies showed progressive elevation of myocardial injury markers, with a marked increase in troponin levels. Hemoglobin levels declined over the course of hospitalization. Metabolic abnormalities became more pronounced, including worsening hypocalcemia with a nadir of 6.4 mg/dL, hyperphosphatemia, hypoalbuminemia, and elevated inflammatory markers (Table 1).

**Table 1:** Laboratory parameters during hospitalization AST: aspartate aminotransferase; LDH: lactate dehydrogenase; CK-MB: creatine kinase-MB; CPK: creatine phosphokinase

Parameter	At presentation	During cardiogenic shock	Early evolution	Reference range
Leukocytes	19.9	11.2	9.6	4.0-11.0 × 10³/µL
Hemoglobin	13.3	10.5	10.6	12.0-16.0 g/dL
Platelets	206	153	133	150-400 × 10³/µL
Glucose	159	92	107	70-110 mg/dL
Urea	39	41	42	10-50 mg/dL
Creatinine	0.6	0.6	0.6	0.6-1.2 mg/dL
Sodium	139	127	133	135-145 mmol/L
Potassium	3.7	3.7	3.3	3.5-5.0 mmol/L
Chloride	101	97	100	98-107 mmol/L
Calcium	8.1	7.0	6.4	8.5-10.5 mg/dL
Phosphorus	-	11.2	2.9	2.5-4.5 mg/dL
Magnesium	1.7	1.7	1.8	1.6-2.4 mg/dL
C-reactive protein	0.5	9.0	-	<0.5 mg/dL
AST	34	105	-	<40 U/L
Alkaline phosphatase	62	48	-	44-147 U/L
LDH	199	652	-	135-225 U/L
Troponin I	0.08	23.46	14.10	<0.04 ng/mL
CK-MB	-	33	26	<25 U/L
CPK total	-	682	504	55-170 U/L
Arterial pH	-	7.43	-	7.35-7.45
PaO₂	-	56	-	80-100 mmHg
SaO₂	-	90	-	>95%

**Figure 1:**
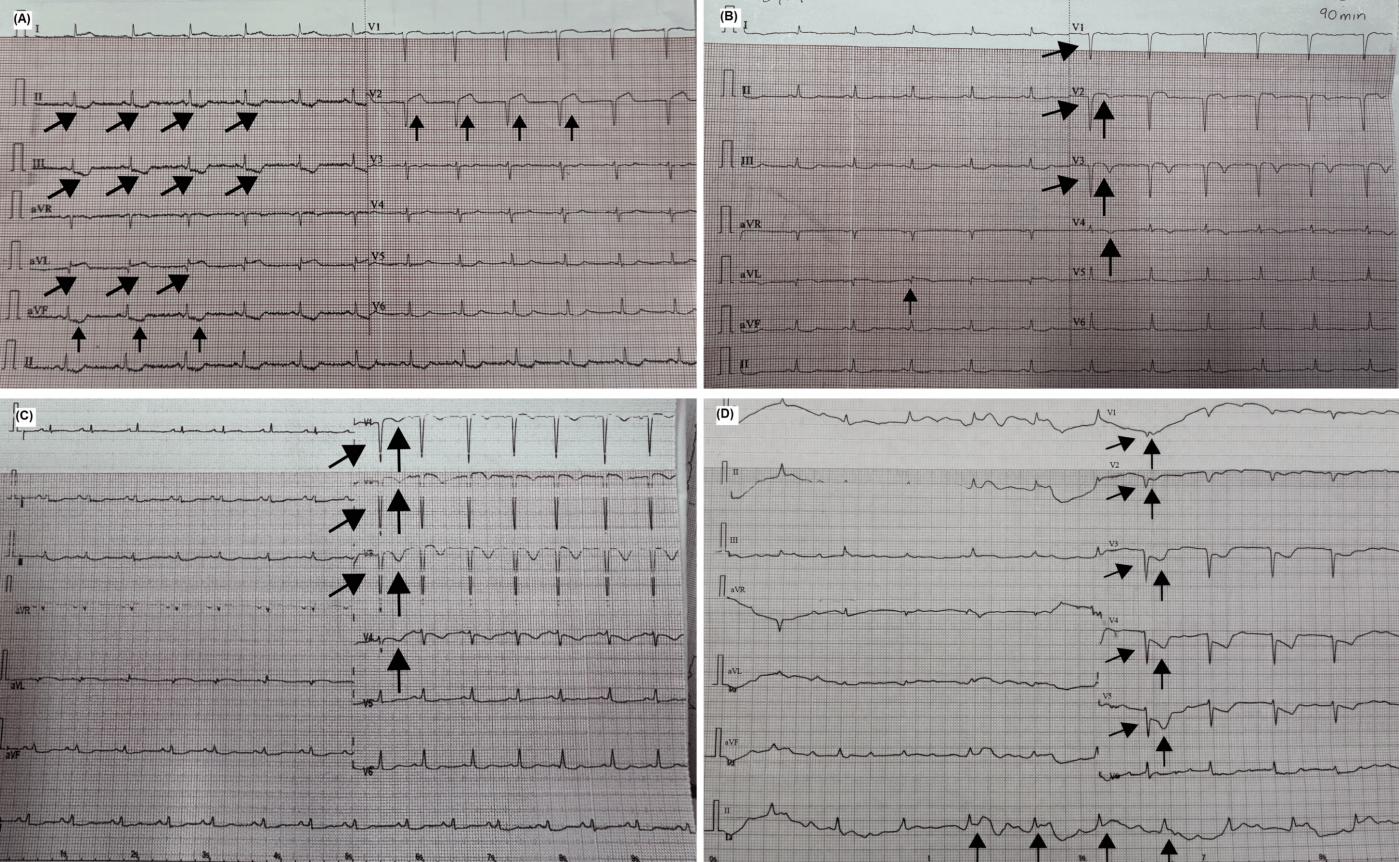
Serial electrocardiographic evolution (A) Twelve-lead electrocardiogram obtained at presentation showing sinus rhythm with T-wave inversion in leads DII, DIII, and aVF, consistent with inferior ischemia. Isolated changes in V2 and aVL are indicated but do not meet criteria for territorial involvement. (B) Electrocardiogram recorded approximately 60 minutes after admission, demonstrating the appearance of pathological Q waves in septal leads and T-wave inversion in anterior leads. (C) Electrocardiogram obtained four days after admission, showing persistent pathological Q waves in septal leads and T-wave inversion in leads V1-V4, consistent with ongoing anteroseptal ischemia. (D) Electrocardiogram recorded on day five, prior to death, demonstrating extensive pathological Q waves in leads V1-V4, T-wave inversion extending from V1 to V5, and ST-segment elevation in leads DII and DIII, consistent with inferior injury. Arrows highlight the principal electrocardiographic abnormalities

Electrocardiographic evaluation at presentation demonstrated sinus rhythm with T-wave inversion in the inferior leads, consistent with acute myocardial ischemia, without initial ST-segment elevation. Serial electrocardiograms revealed dynamic changes, including early development of pathological Q waves in septal leads, followed by progressive ischemic changes in anterior and anteroseptal leads, ultimately evolving toward extensive anteroseptal myocardial necrosis with multiterritorial involvement (Figures 1A-1D). Transthoracic echocardiography was not performed during hospitalization. The patient was admitted during a weekend period when echocardiographic services were not available at our institution, and her subsequent rapid hemodynamic deterioration in the emergency setting prevented delayed imaging evaluation.

Serial electrocardiograms demonstrated dynamic ischemic changes with ST-segment elevation ≥1 mm in contiguous anterior leads (V2-V4), accompanied by evolving pathological Q waves, fulfilling electrocardiographic criteria for ST-segment elevation myocardial infarction in the left anterior descending artery territory. In conjunction with the clinical presentation and the progressive elevation of cardiac biomarkers, these findings indicated evolution from an initial non-ST-segment elevation myocardial infarction to an extensive anterior ST-segment elevation myocardial infarction. Due to limited immediate access to percutaneous coronary intervention, pharmacological reperfusion with tenecteplase (32 mg) was administered (Table 2).

**Table 2:** Timeline of clinical evolution CPAP: continuous positive airway pressure; SCAI: Society for Cardiovascular Angiography and Interventions

Time point	Clinical findings	Electrocardiographic findings	Management	Clinical status
At presentation	Acute retrosternal chest pain at rest, diaphoresis, nausea, vomiting	Sinus rhythm with T-wave inversion in DII, DIII, and aVF; no ST-segment elevation	Initial evaluation and monitoring	Hemodynamically stable
Approximately 60 minutes after admission	Persistent chest pain	Development of pathological Q waves in septal leads and anterior ischemic changes	Continued monitoring and diagnostic reassessment	Ongoing myocardial ischemia
During hospitalization	Progressive clinical deterioration	Dynamic electrocardiographic changes with extension to anterior and anteroseptal leads	Risk stratification and supportive care	Worsening myocardial injury
After pharmacological reperfusion	Acute pulmonary edema and hypotension	Extensive anteroseptal myocardial infarction	Tenecteplase administration, CPAP, vasopressor and inotropic support	Cardiogenic shock (SCAI stage D)
Final evolution	Multiorgan deterioration	Extensive pathological Q waves with inferior ST-segment elevation	Advanced supportive care	Fatal outcome

Following reperfusion therapy, the patient developed acute pulmonary edema and progressive hemodynamic deterioration. She required noninvasive ventilatory support with continuous positive airway pressure, vasopressor therapy with norepinephrine titrated up to 0.09 μg/kg/min, and inotropic support with dobutamine at 2 μg/kg/min. In this context, the clinical picture fulfilled criteria for cardiogenic shock, classified as stage D according to the SCAI classification.

Risk stratification performed during hospitalization classified the patient as Killip-Kimball class IV, with a GRACE score of 122 and a TIMI score of 3. The final diagnoses included cardiogenic shock secondary to ST-segment elevation myocardial infarction, acute pulmonary edema, secondary hypoparathyroidism, moderate hypocalcemia with associated hyperphosphatemia, and hypothyroidism without evidence of clinical decompensation.

## Discussion

Cardiogenic shock remains a devastating complication of ST-segment elevation myocardial infarction, with persistently high mortality despite advances in reperfusion strategies and critical care. Although its epidemiological profile is classically associated with older age, extensive coronary artery disease, and multiple cardiovascular risk factors, growing evidence indicates that severe hemodynamic deterioration may also occur in younger patients, albeit less frequently, and often outside the expected clinical framework.

Large registries and contemporary reviews consistently show that the majority of patients who develop cardiogenic shock are older adults, with age incorporated as a major prognostic determinant in most validated risk prediction models. Wang et al. highlighted that advanced age is a recurrent variable across cardiogenic shock scores, reflecting both accumulated comorbidity burden and reduced physiological reserve [6]. However, they also emphasized that cardiogenic shock frequently develops dynamically during hospitalization rather than being present at admission, with up to 70% of cases evolving after initial presentation. This observation directly aligns with the clinical course observed in the present case, in which cardiogenic shock developed following pharmacological reperfusion rather than at first medical contact, reinforcing the concept of shock as a progressive and time-dependent syndrome rather than a static condition.

The occurrence of myocardial infarction and subsequent cardiogenic shock in younger individuals has received increasing attention over the past decade. Data from the Partners YOUNG-MI registry, as reported by Siddiqi et al., demonstrate that acute myocardial infarction in patients younger than 50 years is becoming more prevalent, particularly among women [7]. However, while young patients may experience substantial myocardial injury, the progression to cardiogenic shock remains relatively uncommon in this age group. This discrepancy suggests that factors beyond chronological age, including infarct size, infarct location, and early post-reperfusion myocardial dysfunction, may play a dominant role in determining hemodynamic collapse.

Consistent with prior observations, severe left ventricular dysfunction, the most common clinical manifestation of cardiogenic shock following acute myocardial infarction, occurs most frequently after anterior infarction involving the left anterior descending artery. Samsky et al. reported that anterior wall infarctions are disproportionately represented among patients who develop cardiogenic shock, reflecting the critical contribution of extensive left ventricular involvement to acute pump failure [2]. In our patient, electrocardiographic findings were consistent with extensive anterior and anteroseptal myocardial injury, which may have outweighed the protective effect typically attributed to younger age.

Importantly, cardiogenic shock may be present at initial presentation or may evolve during the subsequent clinical course. Samsky et al. described cardiogenic shock as a dynamic syndrome driven by loss of contractile myocardial mass, neurohormonal activation, and volume overload, while Wang et al. highlighted that a substantial proportion of patients develop cardiogenic shock during hospitalization rather than at admission, underscoring the evolving nature of this condition [2,6]. In this context, reperfusion therapy does not uniformly prevent early hemodynamic deterioration, particularly in cases of extensive myocardial involvement. A similar progression was observed in this patient, as clinical deterioration developed following reperfusion therapy, emphasizing the need for close clinical monitoring even when reperfusion criteria are met.

Age-based assumptions may further contribute to the delayed recognition of severe complications in younger patients. Zaheen et al. highlighted that myocardial infarction in younger populations increasingly occurs in individuals without traditional modifiable risk factors, particularly among women, and may involve distinct pathophysiological mechanisms [8]. Although nonatherosclerotic causes such as spontaneous coronary artery dissection and myocardial infarction with nonobstructive coronary arteries are more prevalent in younger patients, atherosclerotic plaque rupture remains the dominant mechanism in most cases. This reinforces the importance of maintaining a high index of suspicion for severe ischemic complications regardless of patient age.

Several case reports of myocardial infarction in very young patients, such as that described by Anokhin et al., emphasize the presence of prominent risk factors, including substance misuse or severe dyslipidemia [9]. In contrast, our patient had no identifiable conventional cardiovascular risk factors, highlighting that extensive myocardial infarction and subsequent cardiogenic shock may still occur in young individuals without overt atherogenic profiles. This observation supports the notion proposed by Zaheen et al. that emerging or less apparent risk modifiers may influence disease severity and outcomes in younger patients [8].

Beyond biological risk factors, growing evidence suggests that social determinants of health may significantly influence cardiovascular outcomes. Socioeconomic status, educational level, access to healthcare, and living conditions have been associated with disparities in the incidence, management, and prognosis of cardiovascular diseases. Individuals from socioeconomically disadvantaged populations often experience delayed access to specialized care and reduced availability of advanced cardiovascular interventions, factors that may contribute to worse outcomes in acute myocardial infarction and cardiogenic shock. These structural determinants highlight the importance of considering healthcare access and broader socioeconomic context when interpreting outcomes in severe cardiovascular events [10].

The structured application of the SCAI classification provided a clinically useful framework in this case. Unlike traditional prognostic scores heavily weighted toward age, the SCAI classification conceptualizes cardiogenic shock as a spectrum and emphasizes real-time clinical status and support requirements. As described by Samsky et al., this approach facilitates early recognition, standardized communication, and timely escalation of therapy through serial clinical assessment [2]. In contrast, reviews focused on myocardial infarction in younger populations, such as that by Sood et al., highlight the predominance of epidemiological risk stratification but offer limited guidance for dynamic hemodynamic decision-making in acute shock states [11].

In the present case, classification as SCAI stage D accurately reflected the presence of persistent hypotension associated with acute pulmonary edema, progressive hemodynamic deterioration despite initiation of supportive therapy, and the need for escalation to both vasopressor and inotropic support. These findings were consistent with a deteriorating shock state as defined by the SCAI classification. Laboratory abnormalities, including severe hypocalcemia, may have further contributed to myocardial dysfunction and electrical instability during the phase of clinical deterioration.

Overall, this case reinforces several important clinical messages. First, cardiogenic shock should be recognized as a dynamic and potentially delayed complication of myocardial infarction, including after reperfusion therapy. Second, younger age does not preclude the development of severe hemodynamic compromise, particularly in the setting of extensive anterior myocardial infarction. Finally, reliance solely on age-based risk stratification may underestimate risk in younger patients, underscoring the value of structured clinical assessment frameworks such as the SCAI classification to guide early management.

## Conclusions

This case highlights that cardiogenic shock may develop in very young patients with ST-segment elevation myocardial infarction despite the absence of traditional cardiovascular risk factors and even after pharmacological reperfusion. The distinctive aspect of this report is the occurrence of rapidly progressive cardiogenic shock in a young patient without identifiable cardiovascular risk factors, illustrating that severe hemodynamic deterioration may arise outside the classical epidemiological profile typically associated with this condition.

Extensive anterior myocardial involvement appears to be a key determinant of early hemodynamic deterioration, potentially overriding the expected prognostic advantage of younger age. The clinical course also underscores the dynamic and progressive nature of cardiogenic shock, with deterioration occurring despite reperfusion and escalating vasoactive support, emphasizing the importance of close monitoring after reperfusion therapy.

Structured clinical assessment using the SCAI classification proved valuable in identifying progression to SCAI stage D, facilitating timely recognition of advanced shock and guiding escalation of supportive management. Overall, this case underscores the need to avoid relying solely on traditional risk profiles when evaluating acute myocardial infarction and highlights the importance of maintaining a high index of suspicion for cardiogenic shock even in young patients without conventional cardiovascular risk factors.
